# Learning, working, and teaching non-standard Crystallographic Techniques

**DOI:** 10.1063/4.0001062

**Published:** 2025-10-27

**Authors:** Malliakas D.

**Affiliations:** Northwestern University, Evanston, IL, USA

## Abstract

An overview of learning and teaching experiences using non-standard Crystallographic techniques will be discussed. Past and recent work using 1) multi-dimensional crystallographic methods,1-6 2) total scattering techniques for the elucidation of local and long-range correlations in materials,7-13 3) twinning,14-16 and 4) powder diffraction methods17-21 will be included in this award talk.
